# An Experimental Safety Response Mechanism for an Autonomous Moving Robot in a Smart Manufacturing Environment Using Q-Learning Algorithm and Speech Recognition

**DOI:** 10.3390/s22030941

**Published:** 2022-01-26

**Authors:** Kahiomba Sonia Kiangala, Zenghui Wang

**Affiliations:** 1 College of Science, Engineering and Technology (CSET), University of South Africa, Johannesburg 1710, South Africa; sokiangala@gmail.com; 2 Department of Electrical and Mining Engineering, University of South Africa, Johannesburg 1710, South Africa

**Keywords:** autonomous moving robot, obstacle-free path planning, Q-learning algorithm, reinforcement learning (RL), safety response, smart manufacturing, speech recognition

## Abstract

The industrial manufacturing sector is undergoing a tremendous revolution moving from traditional production processes to intelligent techniques. Under this revolution, known as Industry 4.0 (I40), a robot is no longer static equipment but an active workforce to the factory production alongside human operators. Safety becomes crucial for humans and robots to ensure a smooth production run in such environments. The loss of operating moving robots in plant evacuation can be avoided with the adequate safety induction for them. Operators are subject to frequent safety inductions to react in emergencies, but very little is done for robots. Our research proposes an experimental safety response mechanism for a small manufacturing plant, through which an autonomous robot learns the obstacle-free trajectory to the closest safety exit in emergencies. We implement a reinforcement learning (RL) algorithm, Q-learning, to enable the path learning abilities of the robot. After obtaining the robot optimal path selection options with Q-learning, we code the outcome as a rule-based system for the safety response. We also program a speech recognition system for operators to react timeously, with a voice command, to an emergency that requires stopping all plant activities even when they are far away from the emergency stops (ESTOPs) button. An ESTOP or a voice command sent directly to the factory central controller can give the factory an emergency signal. We tested this functionality on real hardware from an S7-1200 Siemens programmable logic controller (PLC). We simulate a simple and small manufacturing environment overview to test our safety procedure. Our results show that the safety response mechanism successfully generates paths without obstacles to the closest safety exits from all the factory locations. Our research benefits any manufacturing SME intending to implement the initial and primary use of autonomous moving robots (AMR) in their factories. It also impacts manufacturing SMEs using legacy devices such as traditional PLCs by offering them intelligent strategies to incorporate current state-of-the-art technologies such as speech recognition to improve their performances. Our research empowers SMEs to adopt advanced and innovative technological concepts within their operations.

## 1. Introduction

Today’s manufacturing automation trend, the so-called Industry 4.0 or smart manufacturing, is characterized by the application of intelligent technologies and concepts to create smart factories [1]. Under Industry 4.0, modern factories face an increasing level of automation to achieve more efficiency and quality in production processes. One of the key elements of smart manufacturing is the use of robotics technologies in various applications varying from basic to advanced tasks such as parts processing in production lines, product transportation, material handling, logistic warehousing, rescue services, inspection, surveillance, data collection, and more collaborative activities with human operators [2,3,4]. The implementation of collaborative robots (cobots) aims to enhance the cooperation and the shared workforce between humans and robots in smart manufacturing environments [5,6]. Cobots are usually programmed with an extent of autonomy where the robots themselves can perform certain duties without relying on human intervention. When dealing with a moving robot, the concept of autonomy becomes more critical since the robot must safely navigate through the factory from the starting point to the destination by avoiding any obstacle along the way [7]. Obstacle avoidance for moving autonomous robots has been a challenging topic among researchers. One of these researches’ goals is to provide robots with an intelligent behavior system to make appropriate decisions in their working environment. Robot obstacle avoidance falls within the robot path planning procedure [8]. Robot obstacle avoidance has two main categories: obstacle avoidance in a totally or partially unknown environment and obstacle avoidance in a predefined environment [9]. To give robots the ability to make autonomous decisions like human beings, researchers used the great potentials of machine learning to build intelligent robot systems. Machine learning (ML) is a field in the domain of artificial intelligence (AI) [10] that focuses on providing machines (robots) with learning abilities like humans. ML has various eminent applications in the areas of medical treatment [11], speech recognition [12], natural language processing (NLP) [13,14], image processing and signal processing [15].

Reinforcement learning (RL) is a ML approach widely used in robotics to allow autonomous robots to learn obstacle-free trajectories to their destinations in a complex and uncertain environment without necessarily prior knowledge of the working environment. With RL techniques, autonomous robots can improve their performance through trials-and-errors interactions with the environment learning from their experiences to make the most appropriate decision in the future without external assistance. Hence, RL provides the robot a tool to learn and adapt in a dynamic environment [7]. Another essential concept in the human–machine or human–robot interaction in smart factories is speech and language application via speech recognition [16]. Speech recognition [17] is another machine learning function through which machines can understand human speech and languages. Speech recognition eases the communication between humans and robots in a modern factory where the interaction between them is inevitable [18]. Thanks to speech recognition applications, voice instructions and commands can be given directly from human operators to robots.

Our research novelty lies in implementing frontier technologies such as speech recognition and AI in factory floor automation to create an enhanced safety mechanism for small manufacturing plants. Our research is suitable for small manufacturing SMEs intending to adopt advanced technological concepts to improve their operations. We apply a speech recognition process to transform a legacy S7-1200 Siemens PLC into a more intelligent device controlling its output via voice commands to strengthen a factory security interlock. We accomplish voice interfacing to the PLC from the computer microphone without a third-party device. Our voice command to PLC integration contributes in reducing the risks of delayed system stopping response using a central ESTOP far from the operators at the time of emergency. We implement RL to develop a basic safety induction routine for AMR. The safety routine enables co-robot agents to find an obstacle-free path to the closest safety exit in an emergency. We use an RL algorithm called Q-learning to make an autonomous learner (robot) take the most effective path to the closest safety exit. The safety exit can change depending on the robot’s location when the emergency signal is received. The voice command that controls the PLC directly is advantageous for the plant when the operator is far away from the system emergency stop (ESTOP) or stop buttons when the emergency signal needs to be activated. The voice command is an enhancement to an existing emergency stopping interlock.

### 1.1. Motivation of the Study

Safety is a crucial factor in any working organization. In the industrial manufacturing environment, safety has become one of the highest priority areas to ensure an effective production system. Production factories contain several tools, pieces of machinery, and hazardous materials that could easily cause incidents without proper safety measures. An unsafe working environment results in frequent accidents, injuries, and machine failure [19]. One of the well-known emergency responses is to abruptly stop operations using system emergency stops (ESTOPs), often wired in control panels and other strategic locations. Unforeseen disastrous events are almost inevitable, requiring proper emergency responses to be observed by all factories personnel whenever they occur. In a smart factory context, where safety is a cyber-physical production system function [20], all production stakeholders should be involved in safety responses [21]. In an environment where humans and robots collaborate, the emergency procedure should apply to humans and robots. As much as humans go through safety inductions to be instructed on disasters’ best safety measures, robots should comply with the same principle.

Our study proposes a safety enhancement measure for an industrial manufacturing environment in which we equip an autonomous moving robot with an emergency procedure that is activated in case of disaster. We also incorporate a voice command (via speech recognition) as an additional emergency stopping interlock to the central controller (a PLC), improving the emergency stop responses for situations where the operator is far from the ESTOP push button.

### 1.2. Contributions and Limitations

Our primary contribution is developing a safety response system in an intelligent manufacturing environment where humans and machines (robots) collaborate. With the help of a reinforcement learning algorithm, Q learning, we make an autonomous moving robot to go through a “safety induction” to learn by itself the path to the closest safety exit based on its current location instead of relying on a single path to evacuate (that could be very distant from its location at the time of emergency) the place. In an emergency, the shorter the traveling distance to the exit, the better as more incidents could happen along the way. Our experimental factory environment has a total of four safety exits. The destination of the autonomous robot is dynamic and depends on its current location. Our study also contributes to enhancing a PLC emergency stopping interlock by incorporating a voice command as an additional stopping input for the system in case of emergency. The voice command is captured from a microphone directly to the PLC via a python script without any other third-party controller like a Raspberry Pi.

Limitations: We based our study purely on software simulations. It was not tested in a physical manufacturing factory. Our safety response mechanism is suitable for planning purposes before implementation to a real-world application. The voice command to the PLC is an enhancement to an existing ESTOP system. It should not be installed as the only emergency stopping mechanism.

## 2. Previous Works Findings

Several studies have been conducted to implement RL in robotics: Ref. [9] implemented an obstacle avoidance scheme for a mobile robot using an RL algorithm called Q-learning. This study applied two different Q-learning methods with an extensive tuning of the algorithm hyperparameters to obtain the best obstacle avoidance approach. This research was part of robotic competition. Reference [8] developed another study on robot path planning (including obstacle avoidance) using an advanced version of the Q-learning algorithm: an experience-memory Q-Learning (EMQL) through which the agent can receive two types of rewards (static and dynamic rewards). This method improves the robot’s learning ability by utilizing two Q tables that allow the robot to remember a task in case of a change of destination. Another path planning research with Q-learning was carried out by [22], where the robot can safely and effectively navigate in an unknown environment by capturing images (with an onboard robot camera) of the environment and computing the shortest path to the destination. This study applied machine learning to process the captured images. Research by [23] applied RL has in robotics for information gathering in a hazardous gas leakage environment. They implemented an RL framework for the robot to learn how to get gas sources in a known environment. Reference [24] extended RL application to a multi-robot system where several robots are interoperating and moving in a single environment performing different tasks without colliding with one another. They combined Deep Q learning and Convolution Neural Network (CNN) for image analysis to achieve their goal. In order to protect the robot from making fatal decisions during the learning process. Reference [25] defined a new RL approach with two safety algorithms to guide robots when learning their paths. This approach aimed to prevent robots from dangerous states that could destroy them. Reference [2] implements RL to solve a scheduling problem of robots moving from one cell to another loading and offloading production parts. RL allowed creating an effective moving scheduling policy for the robots in real-time to avoid production losses.

The speech recognition field for robotics technology has also been widely exploited with several works such as: Reference [26] who develop an intelligent system that gives robots the ability to detect human emotions by voice input or vision using speech recognition and deep neural networks. Reference [27] created a system to control robot actions and give it instructions at a distance with voice commands. They built the system with a Raspberry Pi as the base controller to process the speech recognition algorithm and send commands to the robot. Another speech recognition for robot command work was conducted by [28]. This study aimed to develop an intelligent robot capable of dressing an individual (disabled people) with speech instructions. Speech recognition was just one of the multiple technologies included in this design, together with gesture and posture recognition. Reference [29] implemented a voice recognition system for an autonomous driving robot in a noisy environment. The system can effectively recognize different voice textures from adults to children despite the noise in the system. They used wireless microphones to capture the voice input and inserted multi-channel voice cancellers to reduce noise. A similar work designed to improve voice command quality in a speech recognition robotic system was conducted by [30]. They improved the output signal of a speech recognizer installed in a control automation environment by applying an intelligent particle swarm optimization approach that tunes the speech recognizer and suppresses environmental noises.

### Previous Researches Gaps Summary

Most previous works on Q-learning (RL algorithm) in robotics have not tackled robots’ safety regarding their working environment. Unlike in traditional factories, where robots are considered static production equipment, their role in a smart factory is more interactive, and they are part of the production workforce. It becomes essential to include them in safety responses in case of emergency. To date, the safety approaches covered in previous research have been on preventing robots from going into dangerous states while interacting with their environment. The fate of the robot in case of plant evacuation or emergency has not been widely explored. Various studies have been done on speech recognition applications in home environments, office environments, and even robotics for communication between humans and robots, but very few have been integrated directly into the process controllers’ structures. A speech command incorporated into the central controller program has the advantage of controlling all other plants’ operations in a single instruction. This feature could be convenient in emergencies. We address these gaps by creating a safety response mechanism (that we consider as a safety induction) for an autonomous moving robot to:

- Find an obstacle-free path to the closest safety exit based on its current location.
- Receive a voice command from the main plant PLC. The voice command is implemented as an additional stopping interlock for the PLC. It means that it can stop all ongoing plant operations in case of emergency.

## 3. Short Theory and Background Overview

### 3.1. Reinforcement Learning (RL)

The reinforcement learning (RL) [31,32,33] algorithm is a ML method created in the early 1990s. The RL algorithm is built based on the enhanced learning principle where an “agent,” the object undergoing the learning process, continuously tries all possibilities of actions and decisions to determine the most optimal one (the best strategy to adopt) [34]. The RL algorithm utilizes five main elements: the agent, the environment, the reward, the state, and the action. In its learning process, the agent performs several interactions with its environment by making some actions that will cause a change of state in the environment and result in a positive or a negative reward (penalty) [35]. Over the years, RL has been the subject of many kinds of research in various applications such as chemical reaction [36], resource management [37], traffic-light management [38], autonomous driving [39], dam management [40], surgery [41] and robotics [42].

Our study focuses on the robotic area where we use RL to equip an autonomous robot, the agent, with an appropriate routine to learn the shortest and safest way to a secure location in case of a disaster that requires evacuation in its working environment. One of the most popular model-free reinforcement learning algorithm we use in this research is the Q-learning algorithm. Model-free algorithms have been successfully implemented in robotics problems because of their ability to reuse information effectively [43].

#### Q-Learning Algorithm

The Q-learning (QL) algorithm is a model-free RL algorithm introduced by by Watkins CJ and Dayan P [44]. It has been developed based on the psychological behaviourism of offering reward or encouragement for good actions and penalties or punishment for wrong actions. The agent performs all possible actions in every state and records the values generated in a Q table. The values in the Q table are computed based on a Bellman Equation [8]. In this research, we implement the QL algorithm to build a safety mechanism for an autonomous robot.

Limitation: It is worth mentioning that the QL algorithm is only suitable for discrete states and actions. Its learning processing time depends on the size of the Q table. The larger the Q table, the longer the learning processing time for the agent [45].

### 3.2. Speech Recognition

Speech recognition is an AI process that converts a voice signal (usually a human voice) into instructions that computers can understand and further manipulate for diverse applications. The voice signal’s conversion involves a transformation of an analog signal (the original voice input) into a digital sequence of sentences or words from the voice instruction [46]. If computer vision [47] is an AI method that allows computers to recognize patterns with the sense of vision or by seeing like humans, speech recognition permits machines or computers to interact with each other and with humans through language or speech.

In recent years, various applications using speech recognition technologies have been developed mainly to substitute traditional input methods like clicking or typing with voice commands. The creation of virtual assistants’ voice-enabled applications such as Google Home assistant, Apple home pod, and Amazon Alexa has become quite popular in home and office environments. However, very few speech recognition applications have penetrated the industrial manufacturing sector. Our study aims to enhance an industrial manufacturing plant’s security by incorporating a voice command as an additional emergency or safety input signal to a traditional PLC. Therefore, we apply the speech recognition process to an industrial manufacturing environment.

Limitation: One of the most significant limitations of speech recognition is that it requires a noise-free input command to carry out its process efficiently. Obtaining a clear input can be challenging because of different language accents and intonations that sometimes the system has difficulty recognizing. Several types of research exist to reduce the noise level in speech recognition input systems. It is not advised to use a speech recognition application as a single input for safety control [48]. In our study, we use the voice command input as an enhancement to an existing safety mechanism.

### 3.3. Programmable Logic Controller (PLC) Functions in the Era of Industry 4.0

Programmable Logic Controllers (PLCs) have been widely used in the industrial sector as the base element for controlling and monitoring field components and production processes. Not many researches talk about this powerful controller [49]. PLCs have been successfully established as the platform of choice for industrial process outline and factory automation conducted by the so-called IEC 61131 standard [50].

Although the industrial and manufacturing sector is undergoing a smart manufacturing revolution, encouraging the utilization of more general-purpose devices, tools, networks, and software, the use of PLCs is not yet ready to vanish. In smart manufacturing, the PLC role evolves from traditional controllers’ tasks to decentralized and integrative functions [51,52]. One of our study goals is to integrate a voice command input via speech recognition to a traditional PLC that initially does not support any voice command. Hence, we transform a traditional isolated PLC system into a smart controller in an Industry 4.0 environment.

## 4. Safety Response Mechanism Methodology

Our experimental safety mechanism for an autonomous robot consists of:

- An automatic path finding section to safety exits in case of emergency: We build this section based on the Q-learning algorithm.
- A voice command input to a Siemens S7-1200 PLC to activate the emergency signal: Speech recognition is the key feature of this segment.

### 4.1. Q-Learning Algorithm to Find Obstacle Free Paths

Q-learning is a reinforcement learning algorithm. One of its working principles is that the agent (the autonomous robot in our research) learns the best policy to adopt for a given scenario based on its interactions with the environment and the rewards gained [35]. According to its current state, a policy in Q-learning is how the agent chooses to behave, react, or make decisions at a given time. Therefore, the policy is a result of the agent’s action and state [50]. A policy can be represented by (1):

(1) β=a|s.

where *a* is the action taken by the agent at a given time and *s* is the state of the agent when taking the action. β formulates the probability an agent takes an action a based on its state *s*.

During the learning process, the Q-learning algorithm computes values generated by pairing each possible action the agent may take based on different states. These calculated values are called Q-values. The agent picks the action that produces the highest Q value. The Q values are stored in a Q Table until the best possible policy is determined by the algorithm [53]. As mentioned previously, the QL algorithm originated from the Bellman equation. The Bellman equation can be applied in a deterministic environment where it is assumed that the agent has 100% chances to go through the desired direction and end up in the desired state when instructed to do so. Euqation (2) is the bellman equation for deterministic environment. The Bellman equation can also be implemented in a stochastic or non-deterministic environment in which there is a probability that the agent may end up in a different state than expected. Euqation (3) represents the Bellman equation for stochastic problems, also referred to as Markovian decision trees [8].

(2) $$G\left(s\right)=\max_{a}\left(r\left(s,a\right)+\gamma G\left(s_{n}\right)\right)$$

where *G*(*s*) is the value obtained at a given state *s*, *r*(*s*,*a*) is the reward obtained by performing an action *a* at a state *s*, γ is an hyperparameter called the discount factor, and $G(s_{n})$ is the value obtained at the next state $s_{n}$.

(3) $$G\left(s\right)=\max_{a}\left(r\left(s,a\right)+\gamma \sum_{s_{n}}P\left(s,a,s_{n}\right)G\left(s_{n}\right)\right)$$

where $P(s,a,s_{n})$ is the probability of getting into the next state $s_{n}$ from the initial state *s* by performing an action *a*. The final Q-learning equation is presented by (4).

(4) $$Q\left(s,a\right)=\left(1-\alpha \right)Q\left(s,a\right)+\alpha [r\left(s,a\right)+\gamma \left(\max_{a_{n}}\right)Q\left(s_{n},a_{n}\right))]$$

where *Q(s,a)* is the Q value computed by an agent performing an action *a* at its current state *s*. α is a Q-learning algorithm hyperparameter that represents the learning rate. Its value is defined in the interval α∈[0,1] and it determines the impact of new information learnt compared to old stored ones. *r(s,a)* is the reward obtained by performing an action *a* at a state *s*. γ is the discount factor. Its role in the Q-learning algorithm is to give importance in future rewards. Like its counterpart α, the value of γ∈[0,1] [9].$Q(s_{n},a_{n})$ is the Q value of the next action $a_{n}$ at the next state $s_{n}$. $\max_{a_{n}}Q\left(s_{n},a_{n}\right)$ calculates the maximum Q value of the next action based on the next state. We can summarize the general operations of the Q-learning algorithm (4) in Algorithm 1.
**Algorithm 1** Q-learning general algorithm for operation summary**Input:** Agent states *s***Output:** Q values  *Initialize Q(s,a) *= 0* and $Q(s_{n},a_{n})=0$*  *Set α and γ*  1. Record a state *s*  2. Choose an action *a* based on the state *s*  3. Examine the reward received *r(s,a)*  4. Record the next state $s_{n}$  5. Find the maximum Q value based on $\left(s_{n},a_{n}\right)$  6. Find the value of *Q*(*s*,*a*)  7. Go back to 1  8. End

The Q-learning algorithm allows our autonomous robot to find an obstacle-free path to the closest safety exit in an emergency. The choice of the safety exit is dynamic and depends on its current location. We create an algorithm (Algorithm 2) to find the route from the current robot location to its destination. This algorithm is a result of our research findings. The computed route should contain the indexes of all the locations through which the robot passes going to the destination. This algorithm utilizes Q values stored in the Q table.

### 4.2. Speech Recognition Process to Activate a Voice Command Input to the PLC

Under the smart manufacturing era, the human–machine interface (HMI) concept has considerably expanded from simple push buttons, light indicators, and graphical user interfaces (GUI) to more advanced interfaces [54] driven by voice and gestures. A new concept called natural human–machine interface (NHMI) is developing enabled by gesture recognition, enhanced reality, and speech recognition [55]. Our research focuses on the implementation of speech recognition to create a new interface for a traditional PLC. The speech recognition principle’s theoretical summary is the ability to recognize and identify patterns of information contained in a speech wave [56]. On the technical side, speech recognition aims to predict the best word sequence *B* based on a speech wave signal *S* and can be represented by (5) for its statistical expression [57]:

(5) $$B^{*}=arg\max_{B}P_{\Psi ,\lambda }\left(B|S\right)$$

where Ψ is the acoustic model and λ is the language model. Equation (5) can be transformed and presented as (6):

(6) $$B^{*}=arg\max_{B}p_{\Psi }\left(S|B\right)P_{\lambda }\left(B\right).$$

where $p_{\Psi }\left(S|B\right)$ represents the probabilistic quantity generated by the acoustic model and $P_{\lambda }\left(B\right)$ is the probabilistic quantity that originated from the language model.

Equation (6) can be further expanded in (7) by assuming that a time sequence *t* is inserted and some observations of the word sequence $b_{t}$ are generated with hidden states $\delta_{t}$ by the hidden Markov models (HMMs).

(7) $$B^{*}=arg\max_{B}P_{\lambda }\left(B\right)\sum_{\delta }\overset{T}{\underset{t=1}{∐}}p_{\Psi }\left(b_{t}|\delta_{t}\right)P_{\Psi }\left(\delta_{t}|\delta_{t-1}\right)$$

The speech recognition system’s first step is to process a feature extraction module, also known as the speech recognition front-end, that produces an acoustic feature. The second step is to transfer an acoustic feature into a language and an acoustic model to obtain the probability of the received initial word sequence. This step constitutes the back-end section of a speech recognition model. The back-end’s output is a sequence of words generated from an acoustic and a language model [57].
**Algorithm 2** Finding the obstacle free path from the current location to the safety exit**Input:** Agent current location *s***Output:** Path to closest safety exit  *Initialize the system, set the Q table*  *Read current location index*  *Set the current location as* start  *Determine the closest safety exit location index*  *Set the closest safety exit as Destination*  **while** (start! = Destination) **do**   1. next location = Location [max Q value (start)]   2. route = start index + next location index   3. start = next location index  **end while**  **return** Route

We use the existing google application programming interface (API) to provide the language and acoustic models for the design of our speech recognition system. The operator’s expected voice command goes through the speech recognition system and is converted into an action enabler for emergency or evacuation cases. We present an outline of a general voice command to controller algorithm, adapted to our voice command input system, in Algorithm 3.

A summary of the procedure of our overall safety mechanism combining the Q-leaning path finding method and the voice command input is presented in Algorithm 4. We assume that:

- θ is the emergency/evacuation signal.
- *X* is the autonomous robot.
- η is a safety exit.

**Algorithm 3** Voice command to PLC input via speech recognition**Input:** Voice command**Output:** Control PLC output*  Import all important libraries (speech recognition and PLC connection)**  Define write and/or read functions for PLC**  Establish connection with the PLC*  **while** (True) **do**   1. Listen to Microphone (sound source)   2. Recognize the language using Google API   voice command == emergency command   Write Instructions to PLC variable  **end while**

**Algorithm 4** Safety mechanism procedure summary
**Input:** Voice command and *X* current location**Output:** System stop and robot path to closest η  1. Activate θ  2. Send θ to *X* from PLC.  3. *X* initiates the safety routine.  4. *X* determines the closest η.  5. *X* finds the obstacle free path to η  6. *X* reaches η  7. end


## 5. Experimental Environment Architecture and Workflow Diagram

**Figure 1:**
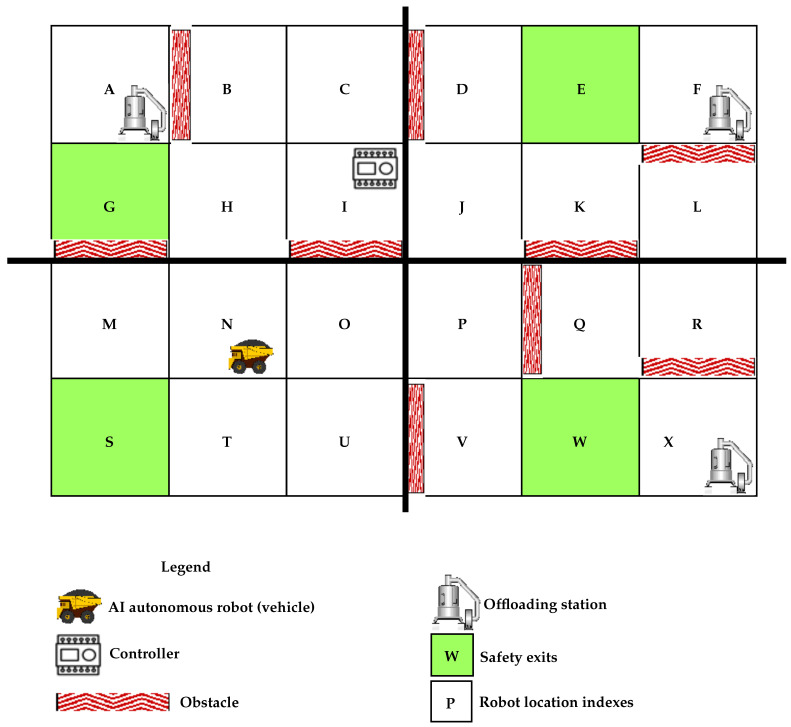
Experimental manufacturing environment for autonomous moving robot.

We present our simple experimental manufacturing location overview in Figure 1 with its legend describing all essential components. The autonomous robot should travel from one location letter to another, avoiding obstacles until it reaches one of the safety exits. In this research, we assume that the obstacles are static. The experimental manufacturing environment has 24 locations (from A to X) with four safety exits (E, G, S, and W). It also has three offloading stations (A, F, and X) to which the AMR moves to offload parts it carries. We assume that the location ’I’ controller is the central S7-1200 Siemens PLC that controls the manufacturing operations. The same controller sends an evacuation signal to the AMR in an emergency. Using Q-learning, our safety enhancement method enables the autonomous robot to choose the correct path to the closest safety exit (E, G, S, or W depending on its current location) when it receives an emergency evacuation signal. We assume that a push-button input generates the emergency evacuation signal to a Siemens S7-1200 PLC or voice command from a microphone directly to the PLC.

**Figure 2:**
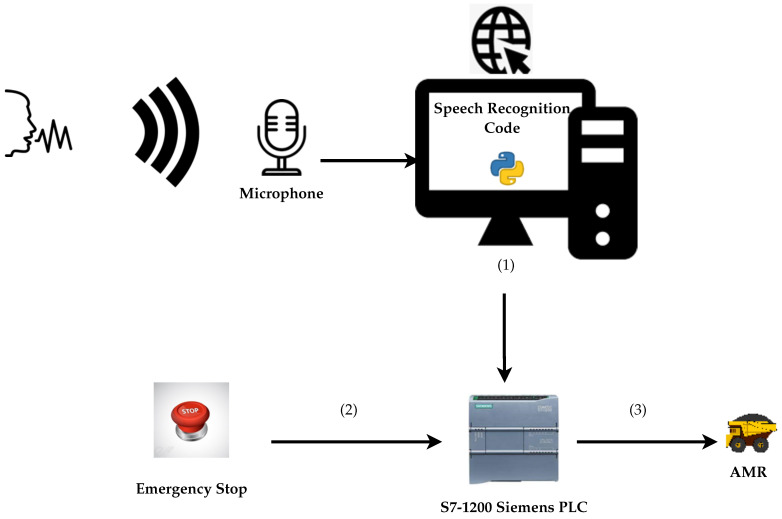
From voice command to PLC emergency evacuation signal.

Figure 2 is a graphical overview of our research work and contribution. It displays the conversion architecture from the voice command to the PLC to activate the emergency signal that triggers the AMR to initiate an evacuation process to one of the four safety exits listed in the above section. (1) represents an emergency voice command signal sent as a PLC input, (2) is an emergency signal originating from the ESTOP button, and (3) represents the emergency plant signal sent to the AMR. As per Figure 2, our research idea and contribution consists of converting a voice command captured by a computer microphone to an evacuation signal sent to an AMR operating in an environment as displayed in Figure 1. The evacuation signal can be given by a plant ESTOP wired as a PLC input. We process the captured voice signal through a speech recognition code, programmed in Python, empowered by Google API (the computer has internet access to integrate the Google API easily). The python script activates a PLC variable programmed to control one of the PLC outputs (sets off the evacuation signal). We assume that the same PLC output instantly triggers the AMR evacuation signal.

**Figure 3:**
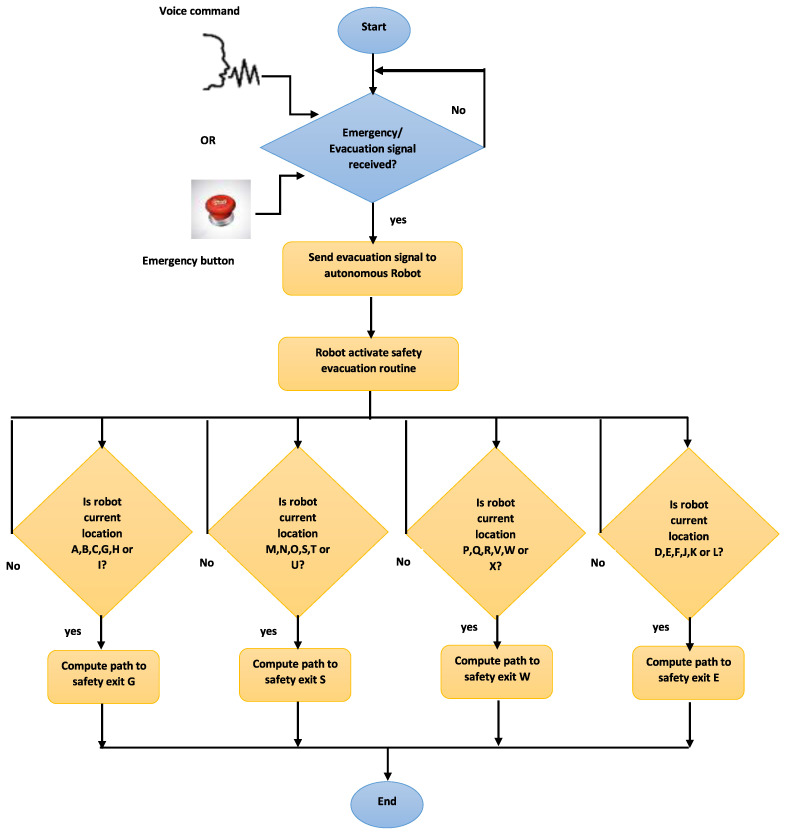
Safety response mechanism flow diagram.

Figure 3 represents another summary of our research work and contribution presented in a workflow diagram with a detailed focus on the AMR response in case of emergency. Figure 3 displays our safety response mechanism once an evacuation signal is received from the voice signal or the plant ETSOP. Our speech recognition code runs an endless loop waiting to receive the voice command to trigger the PLC variable. Once the voice signal arrives, the PLC variable activates an output that sends the robot’s evacuation signal. As soon as the AMR receives the signal, it looks upon its current location to compute the obstacle-free path to the closest safety exit. As part of our assumptions, we considered that the AMR has required functionalities to capture its current location (based on sensors or GPS signal). We divided Figure 1, the experimental manufacturing environment, into four sections, each with its allocated safety exit. This division eases the AMR trajectory computation to one of the closest safety exits. As the AMR always moves from one location index (one letter) to another, its current location will always be one of the 24 locations (no room available for an intermediate location).

## 6. Experimental Results

### 6.1. Experimental Background and Assumptions

- Every industrial plant has or should have a safety protocol to activate in case of emergency. It could lead to evacuating the plant and pressing one of the emergencies stop interlocks (ESTOP or Stop buttons depending on the disaster’s severity) that usually halts the whole system. The stop interlocks are wired inputs of the plant controller (a PLC for this experiment) that are programmed to control several outputs. These stop buttons (including ESTOPS) are connected to the control panels and sometimes on different strategic locations.
*How to palliate to emergencies where none of the operators is nowhere near to the stop buttons?*
  We answer this problem by incorporating a voice command as an additional stop interlock programmed in the PLC. The operator can use the command “emergency” to alt to the system via a microphone. We apply a speech recognition algorithm to convert the operator’s voice command to a PLC’s instruction. A Siemens S7-1200 PLC is used for testing the program via the TIA portal software. We develop the speech recognition code in a python integrated development environment (IDE).
- Significant modern buildings are constructed with different safety exits located at different areas to ensure smooth and efficient evacuations in case of emergencies. It could be more dangerous for people to always go out through a single safety exit that could be far away from their current location, risking getting more injured on the way.
*What happens to autonomous robots in a smart manufacturing environment when a signal to evacuate the plant has been given, and they were busy performing one of their tasks at the plant’s location?*
  We address this problem by implementing the same principle of decentralized safety exits for an autonomous robot. We simulate different safety exits for the autonomous robot based on its current location, and we apply the Q-learning algorithm for the robot to learn the obstacle-free path to the closest safety exit when an evacuation signal is received. We, therefore, reduce the risk of undesired incidents on the robot in an emergency evacuation. We use python to program our Q-learning method for safety exits.

We make the following assumptions:

- In the design of our safety procedure response system, we assume that the autonomous robot has been equipped with onboard means like sensors and cameras to interact with other field components (such as the main plant PLC) and knowledge of its current location.
- We also presume that the microphone used to capture the voice command is powerful enough (wide range) to detect the operator’s emergency command from a long distance and has a noise suppression scheme.
- Another assumption we make in this research is that the autonomous robot has a designated route that does not interfere with human operators’ paths.
- We assume that during the evacuation procedure, the AMR only encounters static obstacles. All other moving obstacles have been stopped accordingly due the state of emergency.

### 6.2. Finding Robot Paths to the Closest Safety Exits Using Q-Learning

From the simulated plant environment layout in Figure 1, we use the Q-learning algorithm to determine the path to the closest safety exit for the autonomous robot depending on its current location in case of an emergency signal that requires a complete exit. Finding the correct paths to the emergency exits include avoiding fixed obstacles in the plant. At the beginning of our design, we assume that the autonomous robot does not know anything about the environment. We divide the plant floor into four segments having each a safety exit of its own. The four segments and their safety exits are as follows:

- For segment 1—locations A, B, C, G, H and I: safety exit G
- For segment 2—locations M, N, O, S, T and U: safety exit S
- For segment 3—locations P, Q, R, V, W and X: safety exit W
- For segment 4—locations D, E, F, J, K and L: safety exit E

One this design’s vital steps is to translate every detail of the experimental plant environment, R, into a matrix of size m x m, with m the plant’s number of locations. The Q-learning algorithm is applied in this matrix format. As per Figure 1, the features of our experimental environment are as follows:

m={A,B,C,...,X}=24

$$R_{size}=24\times 24$$

**Figure 4:**
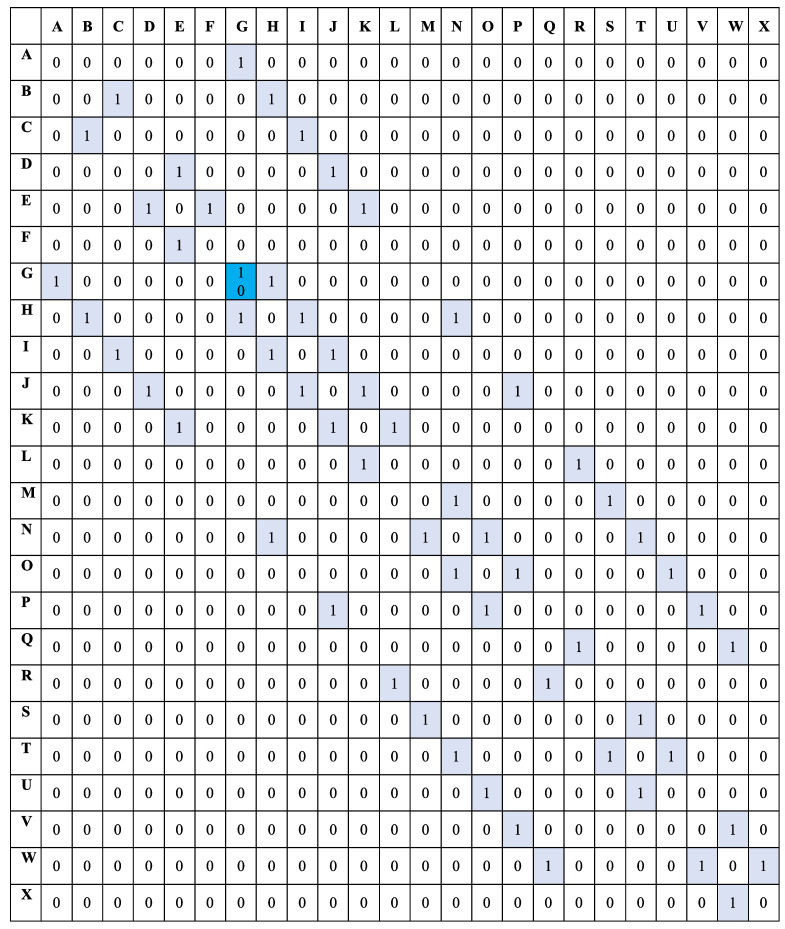
Experimental environment matrix for segment 1 before Q-learning.

Figure 4 is the corresponding environment matrix for segment 1 before applying the Q-learning algorithm. In the matrix R, a ’1’ signifies two neighboring locations, in the intersection of a row and a column, with no obstacles. A ’0’ means that two locations (row and column) do not share a border or have an obstacle between them. A different value such as ’10’ represents the location designated as the safety exit for a specific segment. The value to insert in the safety location is arbitrary and must be greater than 0 and 1. Representing the working environment by its corresponding Q-learning matrix is crucial in generating the obstacle-free path to the desired safety exit. In other words, Figure 4 encrypts locations of our experimental manufacturing environment into a language that the Q-learning algorithm can interpret and use.

**Figure 5:**
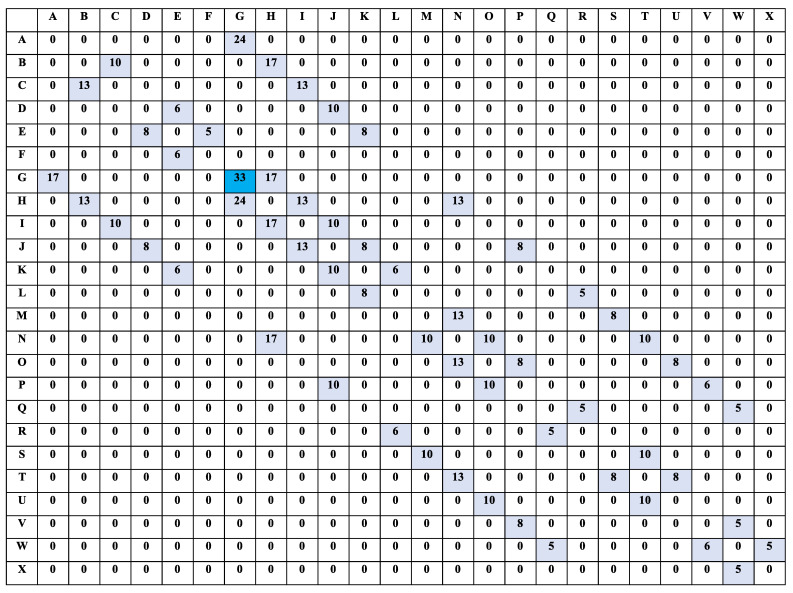
Autonomous robot Q Table for safety response in segment 1.

After applying the Q-learning algorithm to the matrix R, we obtain different reward values for each neighboring location. For segment 1, the safety exit location G has a higher reward value of 33. When moving from one location to another, the autonomous will use the path that gives the highest reward until it reaches the safety exit. From the Q-learning algorithm Equation (4), we chose the Hyperparameters’ following values: α=1 and γ=0.7. After computing the Q-learning reinforcement learning, Figure 4 is converted to Figure 5. Figure 5 is another significant step for the computation of the AMR obstacle-free path. It contains reward values that control the robot path selection based on the RL principle. The AMR chooses the location routes that give higher rewards.

Values in Figure 5 are the rewards given to the autonomous robot for each location visited. The number 33 is the highest reward at location G, the safety exit. We apply the same principle for the three other segments where the main difference is the safety exit location. The safety exit moves from G to S for the second segment, from G to W for the third segment, and from G to E for the fourth segment. From the experimental environment in Figure 1, the rewards values from one location to another for segment 1, based on values in Figure 5, are:

- From A to B or from B to A—Reward value 0. The reward value is 0 because of the obstacle between the two locations
- From A to G—Reward value 24.
- From G to A—Reward value 17. The difference in reward is because going from G to A, the robot moves away from the safety location (G) but from A to G, it moves closer hence a higher reward.
- From B to C—Reward value 0.
- From C to B—The difference in reward is because going from B to C, the robot moves away from the safety location (G) but from C to B, it moves closer hence a higher reward.
- From C to D—Reward value 0. The reward value is 0 because of the obstacle between the two locations.
- From C to I—Reward value 13.
- From I to C—Reward value 10. the difference in reward is because going from I to C, the robot moves away from the safety location (G) but from C to I, it moves closer hence a higher reward.
- From I to J—Reward value 10.
- From I to O—Reward value 0. The reward value is 0 because of the obstacle between the two locations.
- From I to H—Reward value 17.
- From H to I—Reward value 13. The difference in reward is because going from H to I, the robot moves away from the safety location (G) but from I to H, it moves closer hence a higher reward.
- From H to N—Reward value 13.
- From H to G—Reward value 24.
- From G to H—Reward value 13. The difference in reward is because going from G to H, the robot moves away from the safety location (G) but from H to G, it moves closer hence a higher reward.
- From G to M—Reward value 0. The reward value is 0 because of the obstacle between the two locations.

**Figure 6:**
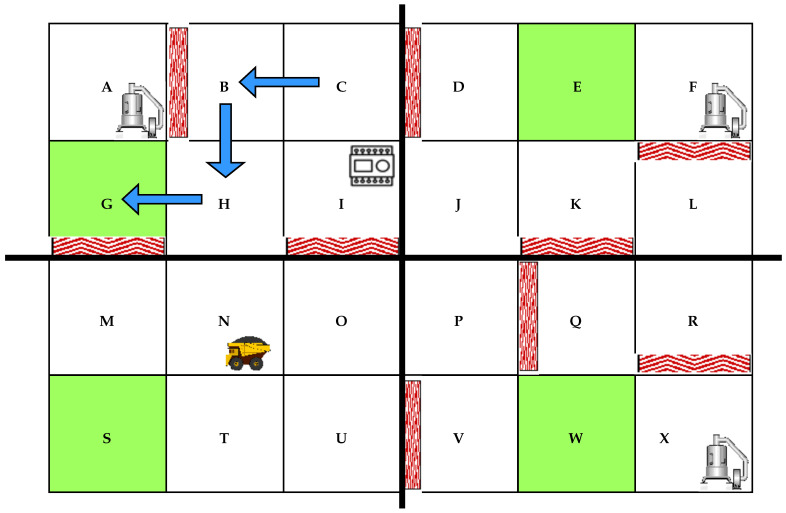
Graphical robot selected path from C to safety exit G.

When learning the best path to the closest safety exit, the robot cumulates the best possible rewards to get in each location until it reaches the destination. We implement Algorithm 2 in our python code to display paths selected by the autonomous robot based on any location it finds itself when the central controller receives the emergency exit signal. The simulation of the current location is done by receiving an input (one of the location letters) and observing the path displayed by our code after computation. This procedure is how the autonomous robot determines the path to the closest safety exit using Q-learning. The Q Table values in Figure 5 are critical elements of the algorithm operation. For an input location (variable loc) equals to location ‘C’, a path to the safety exit ’G’ is set forth. Figure 6 is the graphical representation of the generated path. The same simulation can be tested for any of the 24 locations of the environment. A summary of Algorithm 2 results for location C from the Q Table in Figure 5 can be described as follows: from row C, the highest reward (maximum reward) in line is 13 for both columns B and I. In other words, the autonomous robot can choose to go through location B or I on its way to the safety exit G. Since B is the first column it encounters in the Q table, it chooses B as the following location after C the starting point: C-B. After designating B as the following location, B becomes the new row in which the maximum reward (highest reward) value is selected. As per Figure 5 the highest reward in row B is 17 from column H. H is, therefore, the following location after B: C-B-H. The exact process is applied to H until the autonomous robot reaches the destination, the safety exit, G: C-B-H-G.

The programming code portion to display the robot path from C to G can be summarized as Listing 1:

**Listing 1.** The programming code portion of the robot path from C to G.
**if** loc == ’A’ **or** loc == ’B’ **or** loc == ’C’

**or** loc == ’G’ **or** loc == ’H’ **or** loc == ’I’:

  **print**(route(loc,’G’))

**elif** loc == ’M’ **or** loc == ’N’ **or** loc == ’O’

**or** loc == ’S’ **or** loc == ’T’ **or** loc == ’U’:

  **print**(route(loc,’S’))

**elif** loc == ’P’ **or** loc == ’Q’ **or** loc == ’R’

**or** loc == ’V’ **or** loc == ’W’ **or** loc == ’X’:
  **print**(route(loc,’W’))

**else**:

  **print**(route(loc,’E’))

[’C’, ’B’, ’H’, ’G’]


### 6.3. Using a Voice Command to Control a Siemens S7-1200 PLC Emergency Stop Interlock

An emergency stop interlock is usually programmed in a controller to abort a system’s operation in case of emergency or unforeseen events requiring an immediate stop. In a traditional installation, a stop button or an emergency stop button is wired in the control panel where the operator can stop operations whenever required. For more flexibility in the safety measure, if the operator is far away from the control panel in an emergency, we add a voice input command directly to the PLC controller as an additional interlock to stop the system. Traditional PLCs still operational in several plants have not been designed to receive voice command inputs. Usually, third-party controllers are needed to receive the voice input and interact with the PLC. In this research, we do not use any external controller to receive the voice command; we compute the speech command from the microphone straight to the PLC via a python speech recognition code. We use an S7-1200 Siemens PLC to receive operators’ instructions from a microphone and control its outputs relays with no voice commands capabilities. A simple ladder logic program is written in the Siemens PLC for the stopping and starting interlock. We explain the function and the role of basic required variables for the stopping and starting interlock in Table 1. We performed voice command testings on a physical S7-1200 Siemens PLC hardware to control its outputs.

The start and stop interlock is usually written in a “latch” programming format where a push-button signal (ON-OFF) starts the system (via M0.0), and the outputs (Q0.0 and Q0.2) remain activated until one of the stop commands is activated (M0.2 or M0.1). Necessary experimental settings for the S7-1200 PLC are available as supplementary information. These settings can be implemented to replicate the processor adapted to achieve similar results.

We translate the voice command into an input to the PLC via a speech recognition python code (using a google Application Programming Interface (API)) and a python to siemens PLC library. The speech recognition library converts the voice signal from the microphone into a text that we manipulate in our code to set the PLC variable M0.1. The supplementary article information provides the required settings to achieve a successful speech recognition command to the S7-1200 PLC in the python code.

We present in the article’s supplementary information a representation of a healthy running system monitored from a live S7-1200 PLC when no stop signal is received as well as an explanation summary of each variable’s value in a healthy running system. In case of a fault that requires an emergency shutdown, the voice command can be given via the microphone and directly affect the PLC code. A summary of the variables’ updated values after the stop voice command is presented in Table 2. If we assume that the voice input command is also programmed as a plant evacuation signal, the PLC that controls operations in the whole plant sends a signal to the autonomous robot (via Q0.2 turned OFF) to start the emergency procedure finding their closest path to the safety exit.

### 6.4. Results Summary and Discussion

Our study was about providing additional practical measures to enhance the safety procedures of a smart manufacturing plant. Safety in a manufacturing plant remains a priority for every organization to ensure a smooth run of their production activities [58]. In an era of smart manufacturing and digitalization, the word safety is no longer limited to employees or operators working in the plant but to machine interacting and collaborating with them daily, such as collaborative robots (cobots) and autonomous robots. Firstly, we tackled the safety of autonomous robots moving in a manufacturing plant to perform specific tasks, such as offloading parts to different working stations, by implementing the robot’s Q-learning algorithm to learn the best trajectory to the closest safety exit in case of disaster. Secondly, we incorporated a voice command, with the speech recognition algorithm, as an additional interlock to stop the operation of a plant directly via its control PLC in case of an emergency.

The Q-learning algorithm allows the autonomous robot to learn the most efficient route to the desired target by accumulating the highest rewards along the way. The target has the highest reward. Our experimental environment has twenty-four locations (from A to X) through which the autonomous robot travels. As a strategy to find the closest safety exit for the robot, we divided the environment into four sections where each has its safety exit. In that way, the robot does not have to travel very long distances to get to a designated safety exit when an emergency signal is received from the central controller. During the learning phase, the four targets have the highest reward for the autonomous robot to find its way to any of them depending on its current location. We assigned every location to its specific safety exit. Our Q-learning method’s simulation results demonstrate that an autonomous robot, on its own, finds the best path to the closest safety exit from any location it is based in case of emergency. The trajectory computed by the algorithm is obstacle-free. The emergency routine can be activated as soon as the central controller (PLC) sends an emergency signal.

An emergency stop (An ESTOP) button is a critical component of a plant. It is helpful to stop operations in case of emergency or disaster. It is usually wired on the central controller’s panel and bigger plants in different locations to maximize accessibility. Hence, the closer the operator to the ESTOP at the time of emergency, the quicker the response. We implemented the speech recognition algorithm to use a voice command as a stopping input for the plant if the operator is not close to the ESTOP. In this simulation, the word “emergency” is the command that activates the stopping process. We implemented the speech recognition algorithm directly from a python script running in a computer to a Siemens S7-1200 PLC connected in the same network without going via a third-party device such as a Raspberry Pi.

### 6.5. Research Implications

We summarize the implications of our research as follows:

- In society: Some studies [59,60] highlighted that safety measures and procedures in a manufacturing environment have a direct impact on the frequency of accidents in the plant, to the overall production performance (which in return affects the economy) and to the workers’ well-being. Operators are performant in an environment where they feel safe and comfortable working. Our research offers additional practical strategies for manufacturing plants to enhance their safety procedures in an intelligent manufacturing scenario where human operators and intelligent equipment such as autonomous robots are at risk in emergencies.
- In research: On one hand, our study converts the theoretical concept of the Q-learning algorithm into a practical application directed in the area of safety where much more applications can be developed. On the other hand, we make a traditional controller (a PLC) more intelligent by enabling its ability to receive external voice commands using the Speech-recognition algorithm. The research area of speech recognition for PLCs is still quite open and presents many opportunities for the enhancement of traditional processes in an era of smart manufacturing. Reference [60] states that there is a crucial need for more practical research in manufacturing safety in the way people and external factors or situations can influence the overall safety. Our study is a new addition to this research area.

## 7. Conclusions

In this research, we designed an experimental safety response mechanism for an AMR running in a small manufacturing environment. Our safety mechanism allows the robot to generate a trajectory free of obstacles from its location at the evacuation time to the closest safety exit. We implemented the Q-learning RL algorithm for the robot to grasp the best path to one of our four experimental environment’s safety exits through trials and errors. The robot determines the path to its destination by choosing a location with the best rewards cumulating them until it gets to the exit. We also integrated a voice command, developed with a speech recognition system, as an additional stopping interlock for the manufacturing plant PLC (a Siemens S7-1200). The voice command is captured by a computer microphone processed by a python script in a network computer connected to the S7-1200 Siemens PLC to control its outputs with no additional hardware. Our research novelty lies in applying frontier technologies such as speech recognition and AI (RL algorithm) in factory floor automation to create an enhanced safety mechanism for small manufacturing plants.

We intend to adapt and apply our experimental safety mechanism to a real running factory with all the required parameters changes for future works. We also expect to design a safety mechanism for AMR including moving obstacles instead of static as done in this research. Our safety response mechanism could also be adapted to applications where, in their regular routines, autonomous robots can identify the closest charging spots whenever their batteries are running low. Depending on the working environment’s design, the robot could choose to go to the closest charging location instead of aborting its mission or deciding to go back to the source location.

## Figures and Tables

**Table 1:** PLC start and stop interlock variables.

Variable Address	Variable Function
M0.0	This is the start signal for the system. It is usually a ON-OFF action directly
	wired to a PLC input IX.X. For this simulation, we use a memory bit M.
	When the memory bit is ON the system starts if M0.1 and M0.2 are OFF.
M0.1	This is the memory bit controlled externally by our python speech
	recognition code that converts the voice command to a ‘ON’ bit value.
	When M0.1 is ON the system is in stop mode.
M0.2	This is the memory bit controlled by an emergency stop signal. In real
	plant applications it is usually an input signal IX.X. For simulation,
	we use a memory bit variable M.
Q0.0	This is a physical PLC output that controls the plant.
Q0.2	This is a physical PLC output that controls the plants robots.

**Table 2:** PLC healthy running system start-stop interlock variables values meaning.

Variables	Values	Comment
M0.0	0	Because of the latch programming format. M0.0 does not have to remain
		ON (‘1’) for the system to run continuously.
M0.1	1	An external voice command is received to stop the system.
M0.2	0	No stop signal has been sent by the emergency stop button.
Q0.0	0	The plant control signal is stopped.
Q0.2	0	The robot control signal is stopped.

## Data Availability

Not applicable.
